# Self-Efficacy Among Caregivers of People With Dementia and Its Impact on Caregivers’ Health

**DOI:** 10.1093/geroni/igaa057.1155

**Published:** 2020-12-16

**Authors:** Soyeon Cho

**Affiliations:** City University of New York/CityTech, Brooklyn, New York, United States

## Abstract

Self-efficacy is construct which is associated with positive thinking. It has been examined in caregiving studies to alleviate caregivers’ negative health outcomes. However, little is known about Asian American caregivers’ self-efficacy on their psychological and physical outcomes, especially caregivers with people with dementia. Thus, the present study examined self-efficacy of caregivers as a potential mediator in the association between caregiving role captivity and depressive symptoms among older Korean Americans. Data were driven from a cross-sectional study of 175 community-dwelling Korean American older adults (aged 60 and older) in 2019. The direct significant relation between caregiving role captivity and depressive symptoms became insignificant after self-efficacy was introduced, which demonstrates a full mediation effect of self-efficacy. Results suggest that even in the presence of caregiving role captivity, mental well-being such as depression of caregivers can be maintained by having competence in self-management of their own health.

